# Very small embryonic-like stem cells (VSELs) in adult mouse uterine perimetrium and myometrium

**DOI:** 10.1186/s13048-017-0324-5

**Published:** 2017-04-24

**Authors:** Deepa Bhartiya, Kreema James

**Affiliations:** Stem Cell Biology Department, National Institute for Research in Reproductive Health (Indian Council of Medical Research), Jehangir Merwanji Street, Parel Mumbai, 400 012 India

**Keywords:** Uterus, Myometrium, VSELs, Leiomyomas, Hormones

## Abstract

We have earlier reported the presence of very small embryonic-like stem cells (VSELs) in adult mouse uterus along with slightly bigger progenitors termed endometrial stem cells (EnSCs) and their regulation by ovarian hormones thus demonstrating a crucial role played by them during proliferation, differentiation and remodeling of the endometrium. Present study is a brief communication wherein we have examined the effect of higher dose of estrogen (E, 2 μg/day), progesterone (P, 1 mg/day) and follicle stimulating hormone (FSH, 5 IU/day for 5 days) specifically on the myometrium and perimetrium surrounding the endometrium in bilaterally ovariectomized mice. Similar treatment with E & P was recently used in a study published in the journal Nature to study the effect of steroid hormones on hematopoietic stem cells and this treatment regimen helps achieve hormone levels observed during pregnancy. Quiescent spherical stem cells (lacking PCNA expression) with high nucleo-cytoplasmic ratio and nuclear OCT-4A were detected in the perimetrium of atrophied (bilaterally ovariectomized) uterus. PCNA expression was observed after treatment and cells with cytoplasmic OCT-4B were invariably observed in the myometrium. VSELs were clearly visualized after treatment and the effect of P and FSH was more prominent compared to E on the development of myometrium. It is speculated that stem cells with nuclear OCT-4A located in the perimetrium differentiate to give rise to endothelial and myometrial cells with cytoplasmic OCT-4B. Based on the results of present study and published reports showing the presence of pluripotent markers (OCT-4, NANOG and SOX2) in human myometrial side population and expression of particularly OCT-4A in human leiomyomas, we speculate that these nuclear OCT-4 positive stem cells located in the perimetrium are the possible tumor initiating cells leading to the development of leiomyomas rather than the mesenchymal cells which express cytoplasmic OCT-4B.

## Introduction

Recent published data suggests the existence of a primitive and pluripotent population of stem cells termed ‘very small embryonic-like stem cells’ (VSELs) in various adult organs which express pluripotent and primordial germ cells specific markers and exhibit the ability to expand and differentiate into all three germ layers and also give rise to HSCs and germ cells in vitro [1–4]. Nakada et al. [5] studied the effect of estrogen (2 µg/day) and progesterone (1 mg/day) treatment for 7 days on the hematopoietic stem cells (HSCs) and reported that estrogen promotes expansion of bone marrow HSCs selectively in females. They neither sensitized the mice with low dose of estrogen nor used physiological dose of steroids for their study as is usually done to study the effect of hormones on the uterus [6]. In the present study we have investigated the effect of similar higher dose of estradiol and progesterone (which simulate levels achieved during pregnancy) along with FSH (5 IU/day for 5 days) on the mouse uterus. Present study is focused on the effects of treatment on the perimetrium and myometrium. H&E stained uterine sections and immuno-expression of proliferation (PCNA) and stem cell (OCT-4) markers were studied. Techniques like Western or qRT-PCR were not used as they will not provide any additional information. These procedures involve homogenizing the whole uterine tissue and it will not be possible to study specific effects on the uterine myometrium.

Proliferating cell nuclear antigen (PCNA) is a surrogate marker to study mitogenic effect and monoclonal anti-PCNA mouse IgG antibody (P8825, Sigma) was used in the present study to gauge the effect of treatment on proliferation of myometrial and perimetrial cells. Besides we studied whether the treatment affected stem cells activity by immuno-localization of OCT-4. OCT-4 antibody (ab19857, ABCAM, Cambridge, UK, raised from within residues 300 to the C-terminus of human Oct-4) used in the present study allowed identification of both the alternatively spliced isoforms of OCT-4. Nuclear OCT-4A is crucial to maintain pluripotent state and as the cell initiates differentiation, OCT-4 translocates to the cytoplasm (with no biological function) and eventually gets degraded and is lost in differentiated cells [2]. Similar nuclear and cytoplasmic OCT-4 localization (reflecting spliced variants OCT-4A and OCT-4B) in pluripotent and non-pluripotent human primordial germ cells (PGCs) has been reported by others also [7]. They proposed that OCT-4A in PGCs either translocates to the cytoplasm or is attenuated there possibly for degradation as the significance of cytoplasmic OCT-4 is otherwise unknown. Immuno-histochemistry using 3,3’-diaminobenzidine (DAB) was carried out on paraffin sections and deposition of brown chromogen in Hematoxylin counterstained sections allowed localization of specific cell types in a morphological context.

## Materials and methods

The study was approved by institute stem cells and animal ethics committees. Bilateral ovariectomy was performed on 8 weeks old Swiss mice and after 14 days; they were treated with hormones [estrogen (2 μg/day); progesterone (1 mg/Kg) for 7 days or recombinant human FSH (5 IU/day) for 5 days] via subcutaneous injections into the peritoneum for estrogen & progesterone and in the neck region for FSH. These doses of E & P help attain levels similar to those seen during pregnancy [5]. Uterine tissue was collected and appropriately processed for histological studies and immuno-localization for proliferation marker (PCNA) and a stem cell marker (OCT-4). Paraffin blocks were prepared; sections were cut and stained with Hematoxylin & Eosin (H&E) using standard protocols. Sections were viewed and representative areas were recorded using NIKON 90i Bright- field microscope.

### Immuno-histochemical localization of OCT-4 and PCNA

Briefly, the paraffin embedded uterine tissue sections were deparaffinized and incubated in xylene for 30 mins after air drying slides were incubated with 3% hydrogen peroxide (Qualigens, India) in 100% methanol for 30mins in dark after which the sections were gradually hydrated in descending series of methanol to tap water for 5 min each. This was followed by antigen retrieval by immersing the slides in boiling sodium citrate (SSC, Sigma) buffer at pH 6 for 5 mins. After cooling, the slides were washed with 1X PBS buffer for 5 mins. Permeabilization of the sections was done with 0.3% TritonX-100 in PBS buffer for 10mins for OCT-4. Blocking was done with 10% normal goat serum for 2 h for OCT-4 antibody raised in rabbit and along with 10% normal horse serum for PCNA antibody raised in mice, followed by incubation with the primary antibody OCT-4 (1:100) and PCNA (1:3000) at 4 °C overnight. Primary antibody was replaced with blocking solution for negative control. Next day slides were washed 3 times with PBS (5 min each wash) and then incubated with respective biotinylated secondary antibody for 30 mins followed by avidin biotin complex formation step for 30 min (Vectastain Elite ABC kit, Vector Laboratories Inc, California, USA), 3 washes with PBS and then color reaction was done using diaminobenzidene (Biogenex, USA). The slides were then counterstained with Hematoxylin, dehydrated and cover slipped. Representative areas were photographed under Nikon 90i microscope and the data was recorded.

## Results

### Histological studies

Histological studies were carried out to study both myometrium and perimetrium which were visualized as distinct layers in cross-section. Myometrium comprised of smooth muscle cells arranged in a circular manner surrounding the inner endometrium. On the outer side it was covered by the perimetrium (also termed the serosa).

**Fig. 1:**
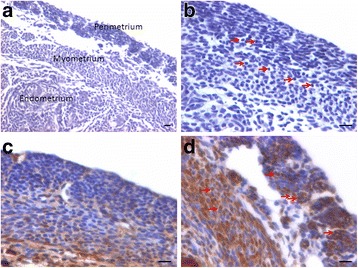
Bilaterally ovariectomized mouse uterine sections after H&E staining and expression of PCNA and OCT-4. **a** Myometrium and perimetrium surrounding the inner endometrium were clearly visualized. They comprised of two distinct layers including outer perimetrium and the inner myometrium. **b** At higher magnification, myometrium comprised rounded cells (*arrow*) as well as sparsely distributed stromal cells. **c** No expression of PCNA was observed whereas (**d**) Nuclear OCT-4A expression (*arrow*) was clearly evident in both perimetrium and myometrium in atrophied uterine sections. Scale bar represents 20 μm

**Fig. 2:**
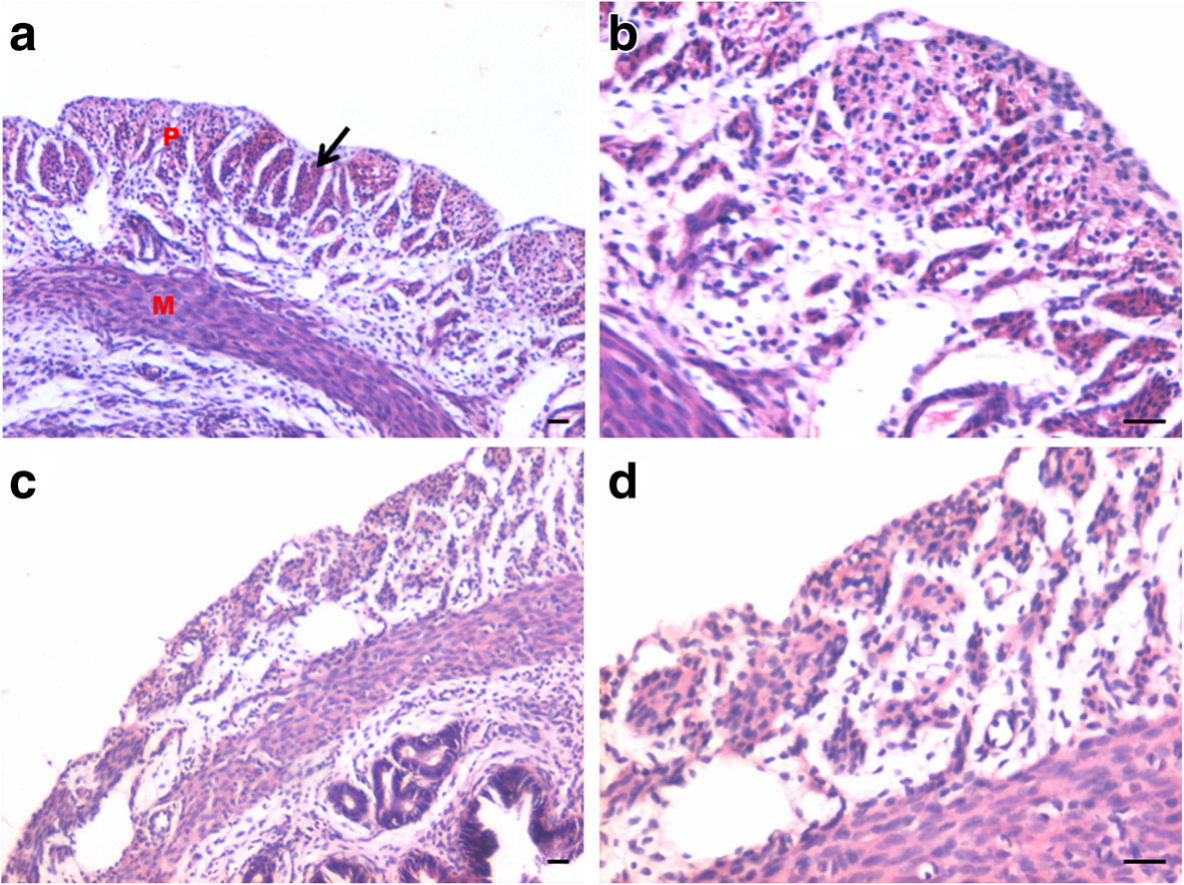
Effect of progesterone and follicle stimulating hormone treatment on mouse uterine myometrium (M) and perimetrium (P). Cellularity was greatly increased after both progesterone (**a**–**b**) and follicle stimulating hormone (**c**–**d**) treatment compared to atrophied uterus shown in Fig. 1. Perimetrial cells were observed to be organized as small islands (*arrow*) and the myometrium was very conspicuous and comprised of tightly woven myoblasts. Note that the spherical cells observed in atrophied uterine sections (Fig. 1) were not observed after treatment with P and FSH. Scale bar represents 20 μm

**Fig. 3:**
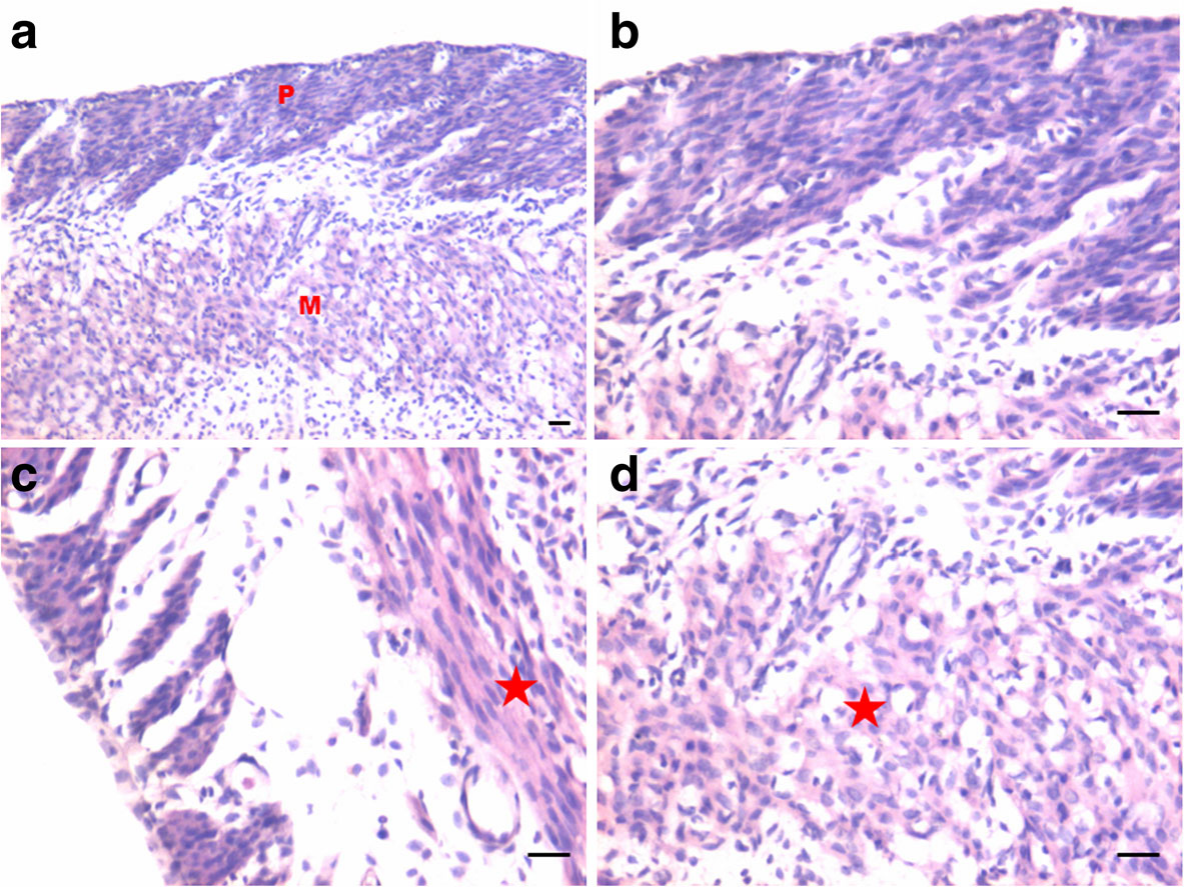
Effect of estrogen treatment on mouse uterine myometrium (M) and perimetrium (P). Estrogen treatment also induced growth of the myometrium and perimetrium but (**a**–**b**) small islands in the perimetrium observed after P and FSH treatment were not prominent after estrogen treatment. Histological appearance of the cells comprising the perimetrium was very different (appeared more undifferentiated) after E treatment compared to those after P and FSH treatment shown in Fig. 2. (**c & d**) Myometrium was also poorly organized comprising loosely arranged cells (*asterix*) compared to that observed after P and FSH treatment (Fig. 2). Scale bar represents 20 μm

In bilaterally ovariectomized mice, both perimetrium and myometrium of atrophied uterus comprised of poorly differentiated cells (Fig. 1a & b). A large number of spherical cells with minimal cytoplasm were clearly visualized (Fig. 1b). The myometrium appeared more organized after both P and FSH treatment (Fig. 2). The spherical cells observed in ovariectomized mice sections were not observed after treatment with P and FSH. These results suggest that the spherical stem/progenitors cells observed in the ovariectomized uterus further proliferate and differentiate into mature cells in response to the treatment. Perimetrial cells were organized as small islands below the outer lining of coelomic epithelium and the myometrium was very conspicuous and comprised of tightly woven myoblasts. Estrogen treatment also induced growth (Fig. 3) but the small islands of cells lining the perimetrium observed after P and FSH treatment were not very prominent in E treated sections. Myometrium was also poorly organized after E treatment compared to after P and FSH treatment and comprised loosely arranged cells (Fig. 3d). Blood capillaries in the junction between perimetrium and myometrium (that were lacking in atrophied uterine sections) became prominent after treatment.

### PCNA immuno-localization in uterine myometrium and perimetrium

**Fig. 4:**
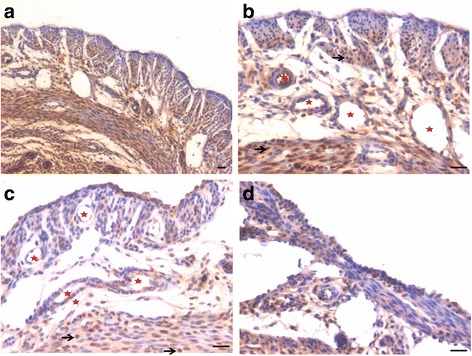
Immuno-localization of PCNA after treatment with progesterone (**a**–**b**) and (**c**–**d**) follicle stimulating hormone. PCNA expression was observed in both P and FSH treated mouse perimetrium but was more in P treated sections compared to after FSH treatment. Cells in both perimetrium and myometrium showed positive expression. Note the development of small blood vessels (*asterix*) lined by a single layer of endothelial cells at peri- and myometrium junction. Excessive non-specific brown color in A is because of endogenous mouse Ig staining since PCNA antibody is monoclonal antibody being used on mouse sections. Scale bar represents 20 μm

**Fig. 5:**
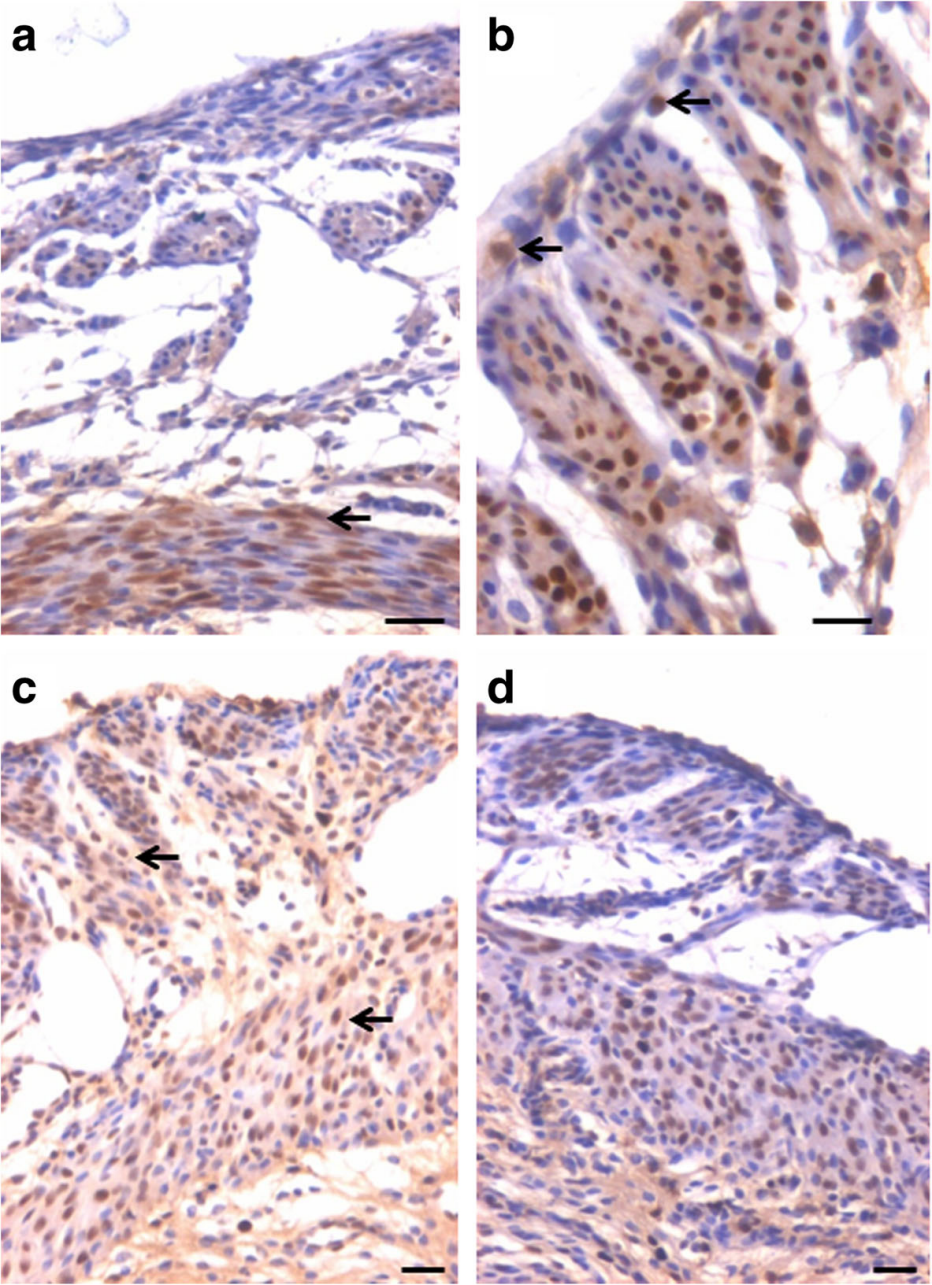
Immuno-localization of PCNA after treatment with progesterone (**a**–**b**) and (**c**–**d**) follicle stimulating hormone. At higher magnification, nuclear PCNA expression was clearly visualized in cells lodged in the perimetrium. Few single cells along the outer lining were also PCNA positive (*arrow*). Progesterone treatment resulted in higher expression of PCNA compared to FSH treatment. Whereas cells in the myometrium showed nuclear to cytoplasmic expression of PCNA. Scale bar represents 20 μm

**Fig. 6:**
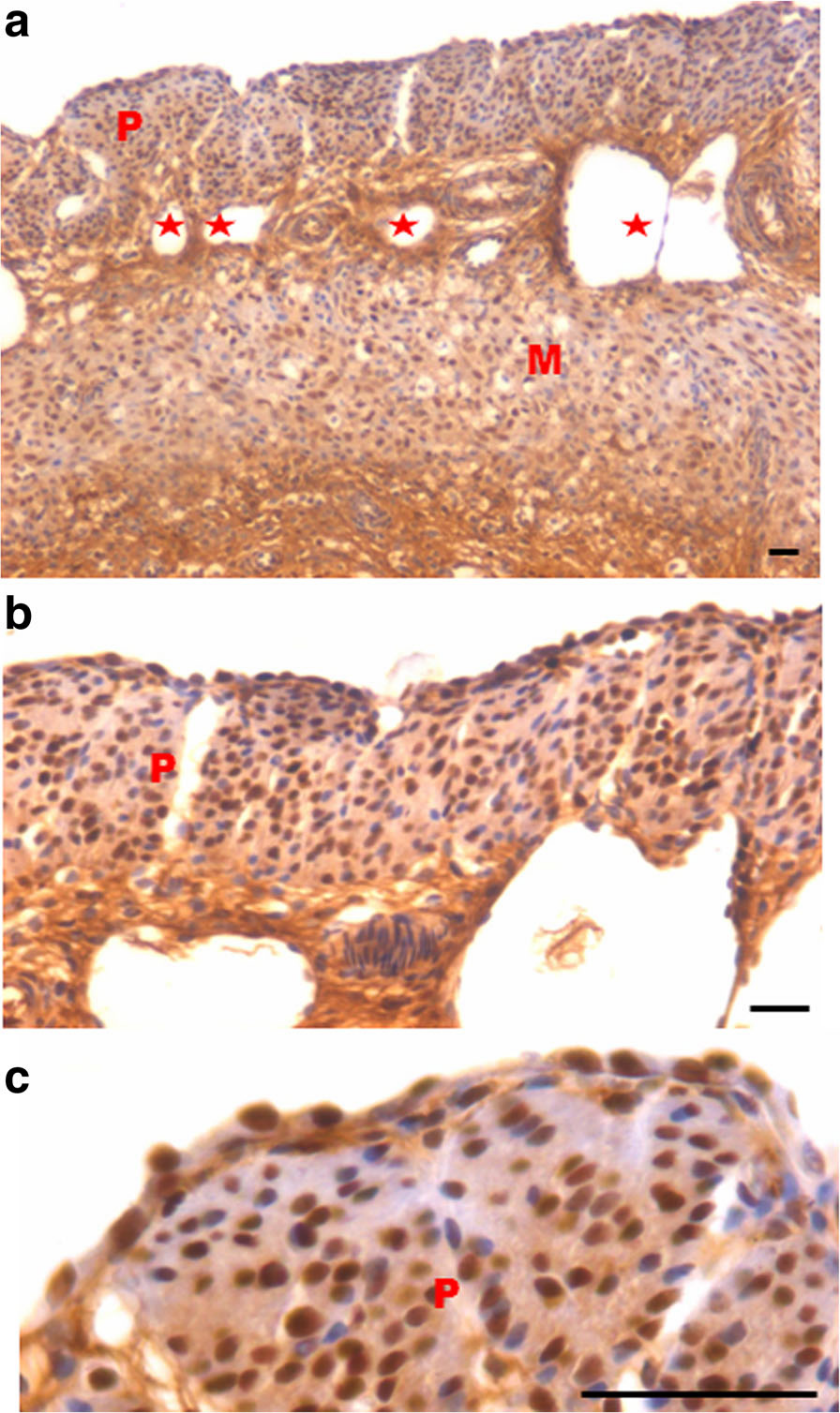
Immuno-localization of proliferation marker PCNA after treatment with estradiol (**a**-**c**). PCNA expression was also noted after estrogen treatment in cells present in both the perimetrium and myometrium. Single cells lining the perimetrium were prominent and expressed PCNA. Small blood capillaries were observed at the junction of peri- and myometrium (lacking in atrophied endometrium, Fig. 1). Excessive non-specific brown color in A is because of endogenous mouse Ig staining since PCNA antibody is monoclonal antibody being used on mouse sections. Scale bar represents 20 μm

**Fig. 7:**
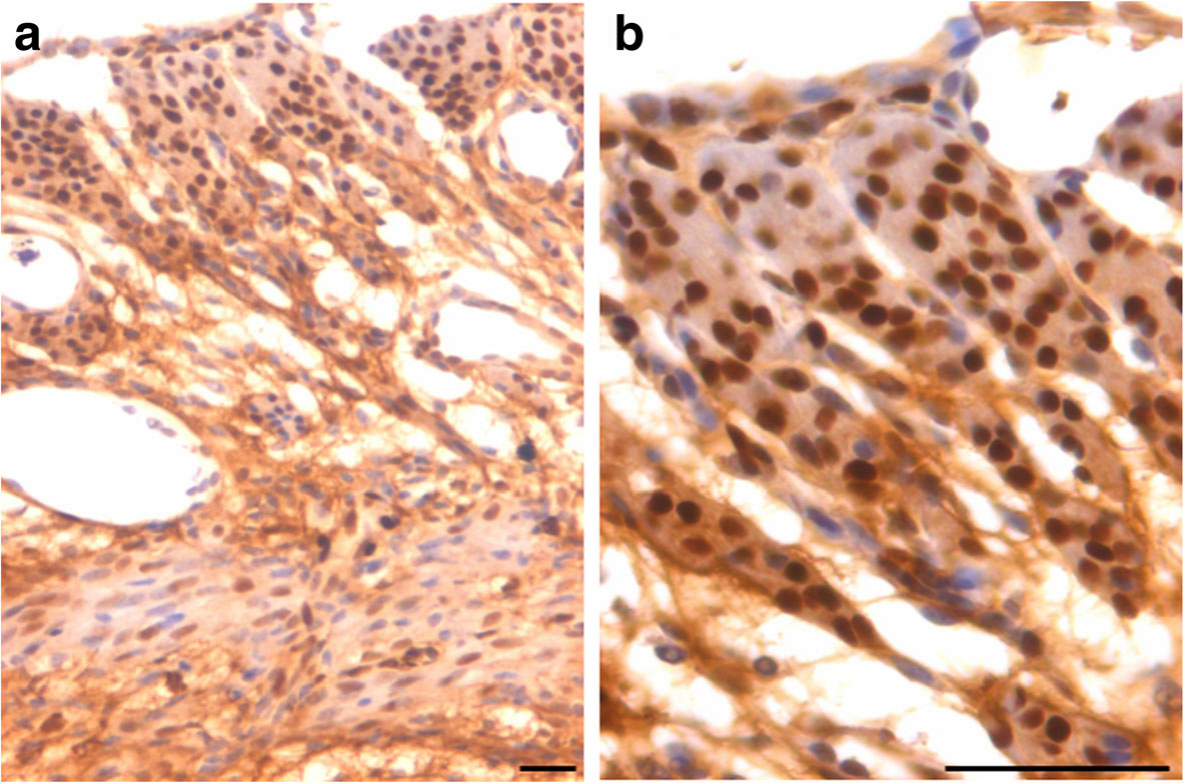
Immuno-localization of PCNA after treatment with estradiol (**a**-**b**). At higher magnification, cells expressing PCNA were visualized in the perimetrium. Only few cells in the myometrium were positive for PCNA. Spindle shaped cells in the myometrium (**a**) were negative for PCNA expression. Scale bar represents 20 μm

PCNA expression was observed in both P and FSH treated mouse perimetrium and was relatively more in P treated sections compared to after FSH treatment (Figs. 4 & 5). Minimal expression of PCNA was observed in atrophied uterus (Fig. 1c). However, both P and FSH treatment resulted in nuclear expression of PCNA in cells of both perimetrium and myometrium. PCNA expression was also noted after estrogen treatment in both cells present in the perimetrium and myometrium (Figs. 6 & 7). Single cells lining the perimetrium were prominent and expressed PCNA (Fig. 6 b & c). In the perimetrium, different patterns of cellular expression of PCNA (nuclear as well as cytoplasmic) were visualized. Few cells with spindle shaped nuclei were negative for PCNA expression. At higher magnification, only few cells in the myometrium were positive for PCNA and majority of cells were negative (Fig. 7).

### OCT-4 immuno-localization in uterine myometrium and perimetrium

**Fig. 8:**
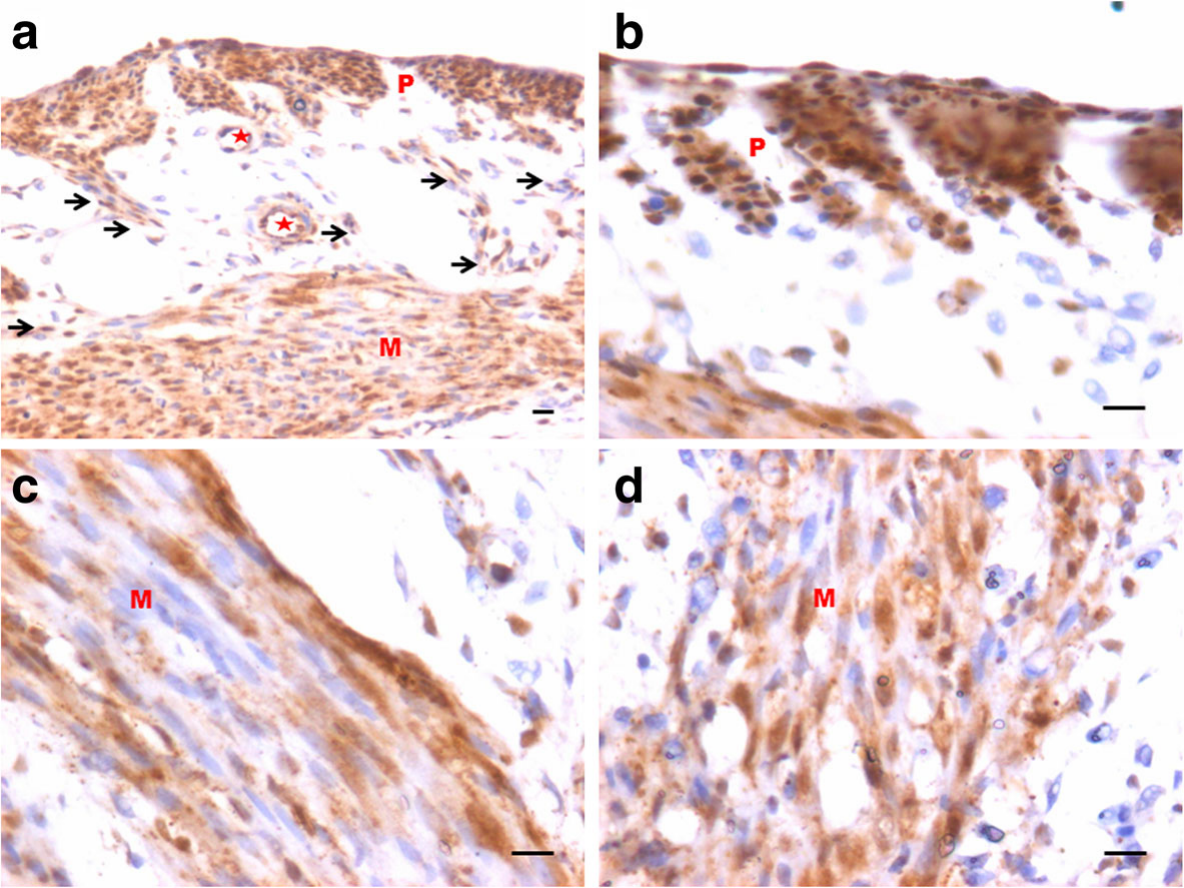
Immuno-localization of OCT-4 in uterine myometrium (M) and perimetrium (P) after treatment with progesterone. OCT-4 expression was clearly observed in the perimetrium as well as myometrium. OCT-4 antibody used in the study detects both cells expressing nuclear OCT-4 (**a**) as well as cytoplasmic OCT-4 (**b**). It is intriguing to note that cells in the perimetrium expressed nuclear OCT-4 (**b**) whereas in the myometrium (**c**–**d**) OCT-4 expression was predominantly cytoplasmic. These observations suggest that the pluripotent stem cells in the perimetrium possibly differentiate into the cells of myometrium. Please note the cells migrating from the perimetrium to the myometrium (*arrow*). Few blood vessels were also observed (*asterix*). Scale bar represents 20 μm

**Fig 9:**
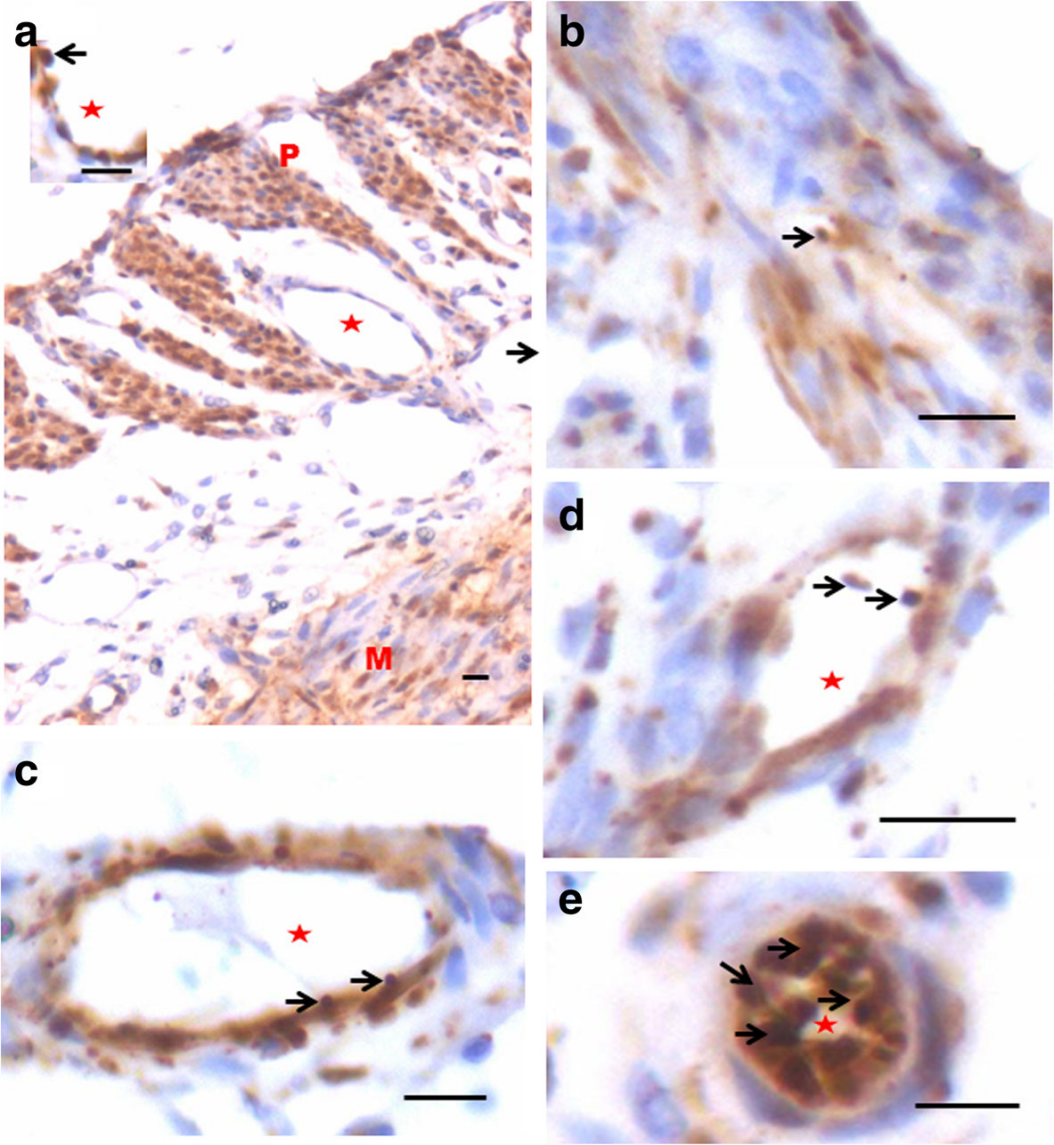
Immuno-localization of OCT-4 in uterine myometrium and perimetrium after treatment with P (**a**-**e**). At higher magnification, few small spherical cells with nuclear OCT-4 (**a**) were clearly visualized (*arrow*). Blood vessels (*asterix*) were visualized in the perimetrium which had spherical cells in their lumen expressing nuclear OCT-4 (*arrow*). Scale bar represents 20 μm

**Fig. 10:**
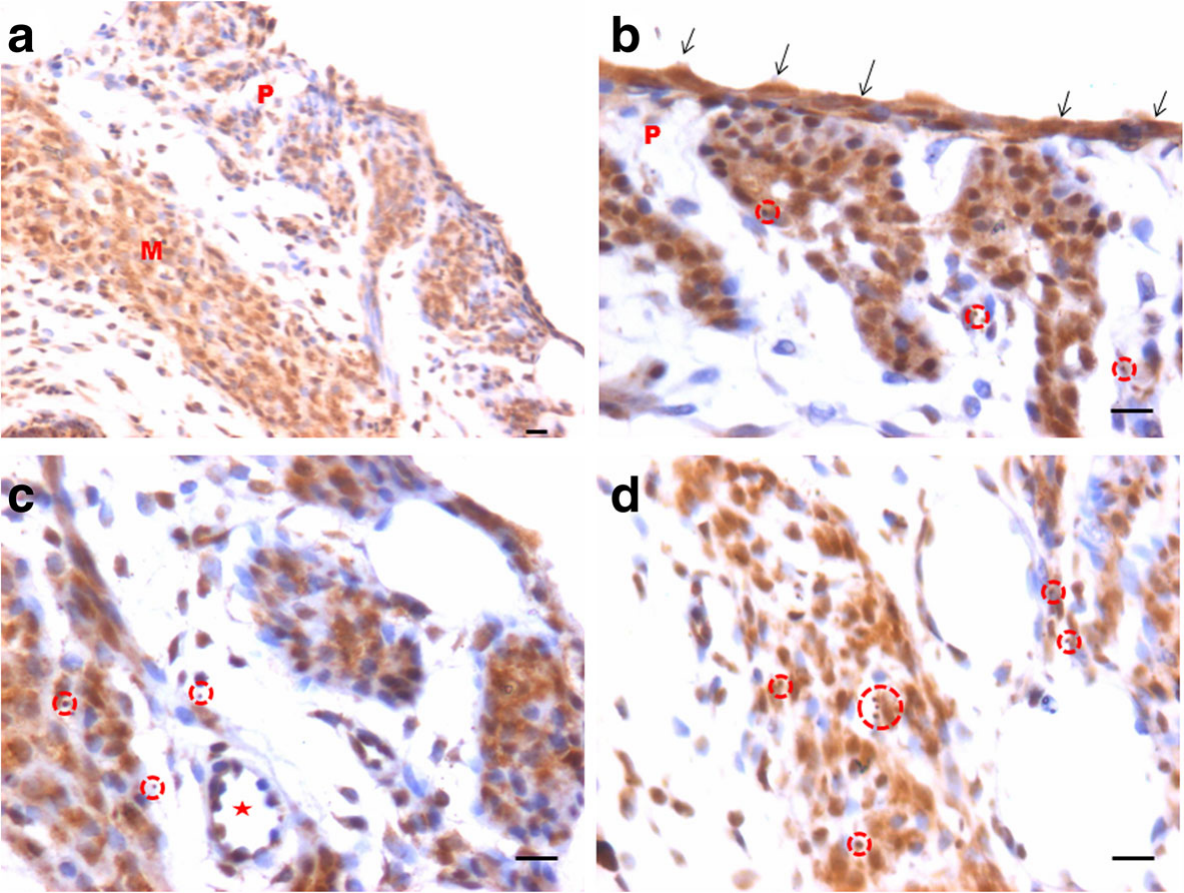
Immuno-localization of OCT-4 in uterine myometrium and perimetrium after treatment with FSH (**a**-**d**). Similar pattern of nuclear and cytoplasmic OCT-4 positive cells in the perimetrium and myometrium were observed as after P treatment. The spherical cells expressing nuclear OCT-4A were clearly visualized at higher magnification (*encircled*). Also cells lining the perimetrium were positive for OCT-4 (*arrow*). Scale bar represents 20 μm

**Fig. 11:**
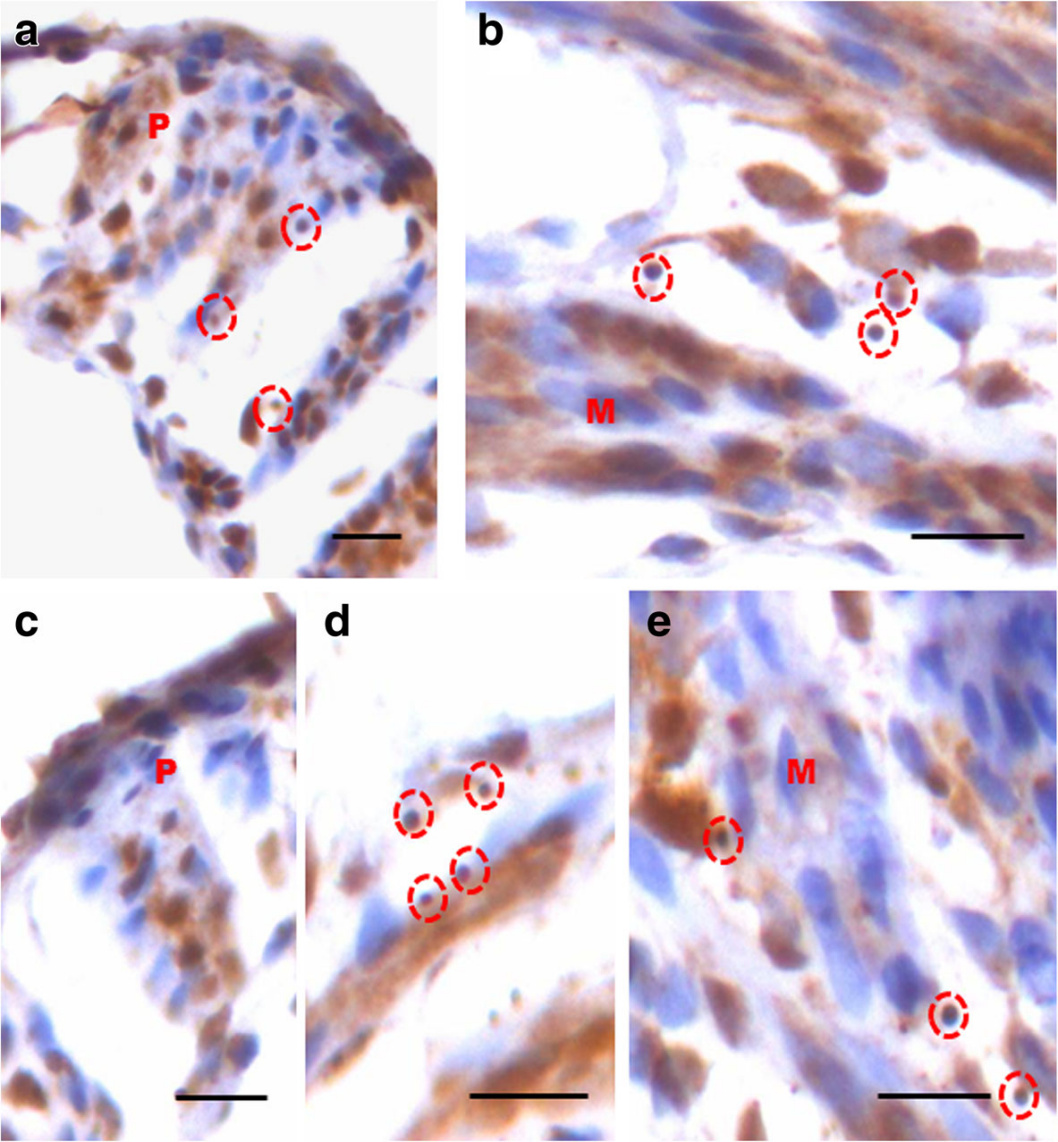
Immuno-localization of OCT-4 in uterine myometrium (M) and perimetrium (P) after treatment with FSH (**a**-**e**). At higher magnification, OCT-4 expression was clearly observed in the perimetrium and myometrium after FSH treatment. Cells in the perimetrium showed cells expressing nuclear OCT-4. In the myometrium, most of cells showed cytoplasmic expression. Interestingly few spherical cells of small size were observed positive for OCT-4 expression (*encircled*). Scale bar represents 20 μm

**Fig. 12:**
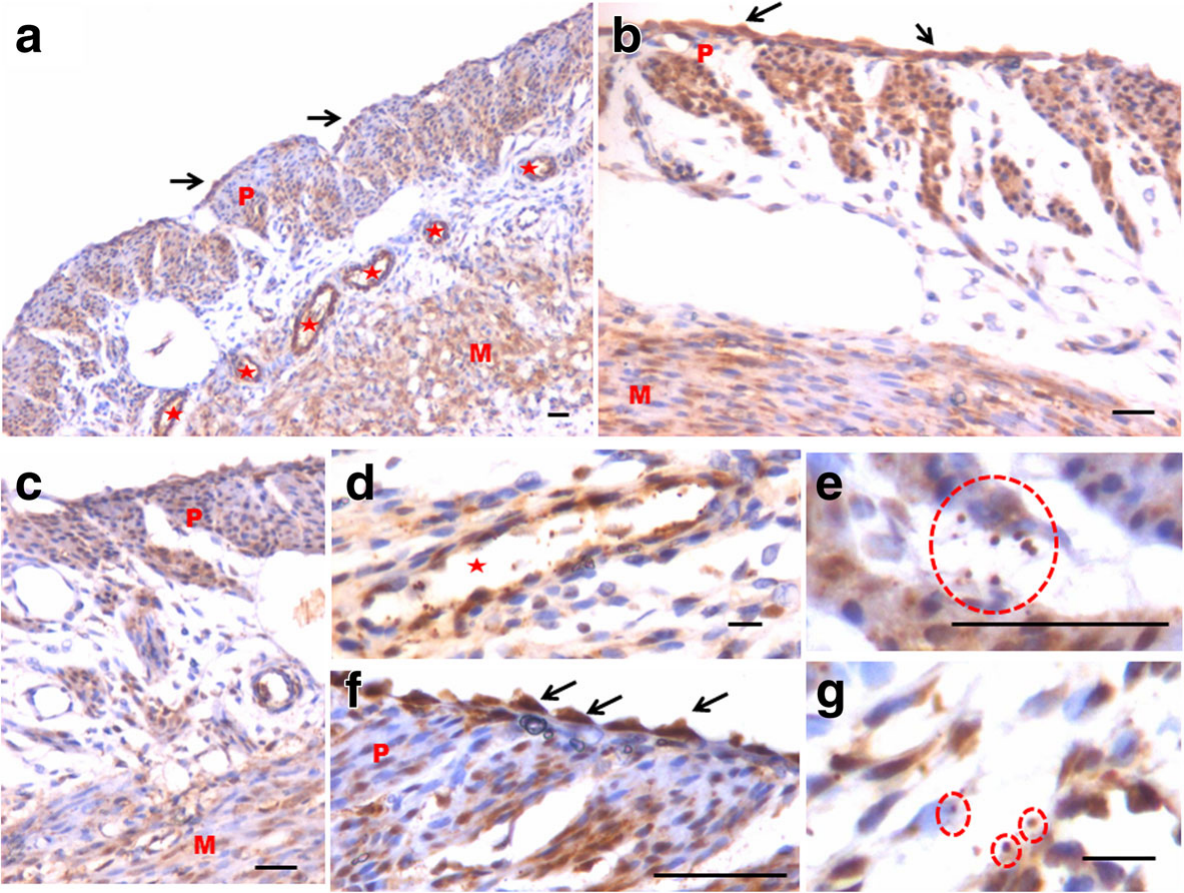
Immuno-localization of OCT-4 in uterine myometrium (M) and perimetrium (P) after treatment with estradiol. With the help of OCT-4 antibody that detects both nuclear and cytoplasmic OCT-4, this figure provides evidence suggestive of pluripotent VSELs differentiate into both endothelial and myometrial cells. **a** At low magnification, OCT-4 expression was noted in cells in the perimetrium, myometrium, coelomic epithelium as well as in the cells lining the blood vessels (*asterix*). **b** Similar distinct pattern of OCT-4 expression including nuclear OCT-4 positive cells in the coelomic epithelium and perimetrium and cytoplasmic OCT-4 in the myometrium was clearly visualized after estradiol treatment. **c** This representative field is suggestive of how nuclear OCT-4 positive cells in the coelomic epithelium/perimetrium differentiate into cells with cytoplasmic OCT-4 that migrate downwards and align as the myometrial cells. Nuclear OCT-4 positive stem cells also differentiate into endothelial cells lining the blood vessels that appear after treatment compared to atrophied uterus (Fig. 1). **d** High magnification of a blood vessel in section with small spherical VSELs expressing nuclear OCT-4 in the lumen. Note that the endothelial cells express cytoplasmic OCT-4. (**e** and **g**) Higher magnification showing small, spherical cells with minimal cytoplasm expressing nuclear OCT-4. **f** High magnification showing coelomic epithelial cells lining the perimetrium expressing nuclear OCT-4 positive cells. Scale bar represents 20 μm

OCT-4 expression was clearly observed in the perimetrium as well as myometrium. OCT-4 expression in uterine sections of atrophied uterus showed an interesting pattern (Fig. 1 d). The atrophied uterine sections were enriched for nuclear to cytoplasmic OCT-4 expressing stem/progenitor cells. P treatment resulted in increased expression of OCT-4 in both perimetrium and myometrium (Figs. 8 & 9). Interestingly, cells in the perimetrium expressed nuclear OCT-4 (Fig. 8b) whereas in the myometrium, OCT-4 expression was predominantly cytoplasmic (Fig. 8 c & d). At higher magnification, few small spherical cells with nuclear OCT-4A were clearly visualized (Fig. 9). Few blood vessels were visualized in the perimetrium which had spherical cells in their lumen expressing nuclear to cytoplasmic OCT-4 (Fig. 9). OCT-4 expression was also observed in the perimetrium and myometrium after FSH treatment (Figs. 10 & 11). Cells lining the perimetrium (coelomic epithelium) and the perimetrium were positive for nuclear OCT-4 (Fig. 10b). In the myometrium, most of cells showed cytoplasmic expression. Interestingly few spherical cells were observed in the myometrium positive for OCT-4 expression (Fig. 10). The spherical cells expressing nuclear OCT-4A were clearly visualized at higher magnification (Fig. 11). Similar expression pattern for OCT-4 was also observed after E treatment (Fig. 12). Blood capillaries were prominent after E treatment and cells lining the perimetrium were positive for nuclear OCT-4 (Fig. 12 b & f). Small nuclear OCT-4 positive cells were observed in the lumen of blood capillaries as well as interspersed among cytoplasmic OCT-4 positive perimetrial/myometrial cells (Fig. 12d, e & g). It was intriguing to note that mesenchymal cells with cytoplasmic OCT-4 (possibly arising by differentiation of nuclear OCT-4 positive cells in the perimetrium) appeared to move down from the perimetrium towards the myometrium (Figs. 7a, 8a, 10a and 12 b & c).

## Discussion

**Fig. 13:**
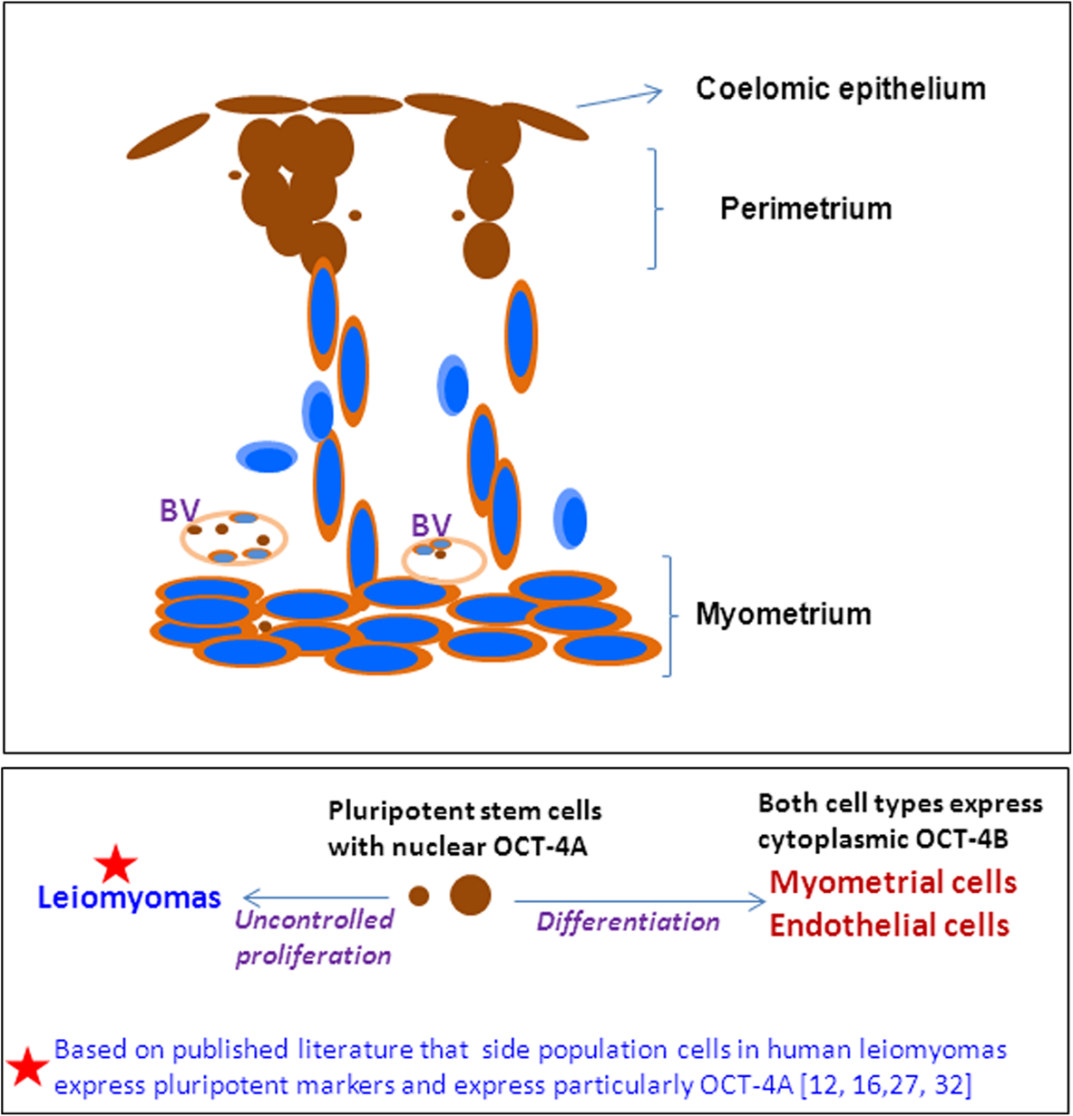
Schematic depicting stem cell biology in uterine myometrium and perimetrium based on our results shown in Figs. 10–12. Brown color depicts OCT-4 immuno-expression. Blue color nuclei depict Hematoxylin counterstained nuclei with absent nuclear OCT-4 expression. Cells lining the surface and in perimetrium express nuclear OCT-4. They are interspersed by small size cells which also express nuclear OCT-4. From the base of the perimetrium, spindle shaped mesenchymal cells emerge which express cytoplasmic OCT-4 (suggestive of differentiation of nuclear OCT-4 positive cells). They migrate downwards and align as myometrium. Few cells with absent OCT-4 expression possibly represent more differentiated state. This stem cells activity in the uterine myometrium and perimetrium became evident only as a result of treatment with higher dose of E, P or FSH to bilaterally ovariectomized mice. Otherwise under normal conditions, these stem cells act in a very subtle manner and have remained elusive so far in the published literature

Present study provides direct evidence for presence of OCT-4 expressing stem/progenitor cells in the atrophied (bilaterally ovariectomized) uterine perimetrium and myometrium. The cells appear undifferentiated and in a quiescent state as evidenced by marked absence of PCNA expression. Treatment with E, P and FSH resulted in increased expression of PCNA and OCT-4 suggesting an activation of stem cells by the treatment. Coelomic epithelial cells lining the perimetrium expressed nuclear OCT-4 as well as PCNA. Perimetrial cells expressed nuclear OCT-4 whereas cells in the myometrium predominantly expressed cytoplasmic OCT-4. Also a distinct population of nuclear OCT-4 positive VSELs was clearly visualized among the mesenchymal cells expressing cytoplasmic OCT-4 (Fig. 13). Results of the present study suggest that the most primitive, nuclear OCT-4A positive cells in the coelomic epithelium and perimetrium possibly differentiate into myometrial cells with cytoplasmic OCT-4. Use of a higher dose of E & P in the present study made it possible for us to uncover the myometrial stem cells for the first time. We have similarly reported existence of VSELs (with nuclear OCT-4) and the progenitors EnSCs (with cytoplasmic OCT-4) in mouse uterus [8] and also in the gonads reviewed in [3]. Various studies provide evidence in support of pluripotent stem cells in the myometrium but have ended up detecting mesenchymal stem cells which are in abundance. Our results provide first direct evidence in support of nuclear OCT-4A positive, small, spherical stem cells with minimal cytoplasm in the mouse perimetrium and myometrium. One could also carry out flow cytometry studies to locate VSELs in SP or in myometrial cells suspension for various cell surface markers as LIN-/CD45-/SSEA-4+ and CD133+ in humans and as LIN-/CD45-/SCA-1+ cells in mice, provided care is taken to always spin cells at 3000 rpm during processing for various experiments (otherwise VSELs will invariably get discarded).

We had recently argued [9] that MSCs could not be the elusive uterine stem cells as suggested by Gargett’s group [10]. However, they [11] were not convinced since uterine VSELs have been reported only in one earlier pilot study [8]. Present study provides further direct evidence in support of VSELs in the mouse uterus and their regulation by steroid hormones. Interestingly the results show more significant effect of P and FSH on myometrial stem cells compared to E. However, further studies need to be undertaken to substantiate these findings. Effect of FSH treatment alone on uterine myometrial stem/progenitor cells was indeed striking and FSH receptor expression has been reported on stem cells in the testis, ovary and the hematopoietic system reviewed in [2].

Stem cells in the myometrium are considered crucial for the extraordinary growth of the uterus to accommodate the growing baby. Two major groups have characterized stem cells in the uterine myometrium including Al-Hendy’s and Bulun’s group [12]. Al-Hendy’s group has reported that Stro-1+/CD44+ cells which are CD45-/CD34-/CD31- and express Oct-4, DNMT3 and c-Kit as possible stem cells in human and rat myometrium and also in fibroids by studying cells in myometrial side population [12, 13]. These stem cells reduce in numbers with age and were increased in numbers after E + P and E treatment while P did not show any significant effect. Developmental exposure to endocrine disruptor resulted in expansion of these cells thereby also increased the risk for fibroid formation. While Bulun’s group [14] have reported CD34+/CD49+ cells in myometrial SP which also express KLF4, NANOG, SOX2 and OCT-4 as the possible myometrial stem cells and also tumor initiating cells. Stem cells have been reported in human myometrial side population by others also [15] as a relatively quiescent population with majority of them being in G0 phase with low expression of ER & PR, grow under hypoxic conditions and abundant expression of OCT-4 & NANOG. Lupicka et al. [16, 17] have reported presence of pluripotent cells expressing OCT-4, NANOG and SOX2 in all the compartment of bovine uterus including myometrium and also in bovine uterus with adenomyosis. Label retaining cells have been reported in beta-catenin deficient mouse uterine myometrium (degenerative uterus with myometrium replaced by adipose tissue) and they also respond to gonadotropins [18].

Stro-1 is considered best marker for mesenchymal stem cells [19] and Mas et al. [12] mention that the cells they isolate also amplify c-Kit, a marker suggestive of differentiated cells rather than stem cells. Moreover, the primers used by them to amplify OCT-4 are for total OCT-4 and not specific for nuclear OCT-4A. They do not mention whether they failed to amplify other pluripotent markers like NANOG or SOX2 or these markers were not studied. It is evident that Al-Hendy’s group has successfully isolated mesenchymal stem cells in the myometrium. Whereas Bulun’s group seems to have enriched a more primitive population expressing OCT-4/NANOG/SOX-2/KLF4 suggestive of pluripotent state. However, it is crucial for Bulun’s group to demonstrate co-expression of CD34/CD49 with pluripotent markers, otherwise their results could also suggest co-existence of two distinct subpopulations of cells (mesenchymal and pluripotent stem cells). The surface phenotype of CD34+/CD49+ has been earlier described on adipose derived stromal cells by another group [20].

Involvement of VSELs in initiating cancers was suggested for the first time by Ratajczak et al. [21] and recently reviewed [22]. Leiomyomas (also known as myomas or fibroids, benign tumors that arise in the myometrium), are most common tumors in women resulting in hysterectomy. They are monoclonal tumors that arise from stem cells lodged in the uterine myometrium [23, 24], have been associated with MED12 mutations and WNT/CTNNB1 pathway is involved in their pathogenesis. Al-Hendy’s group [12] suggested that Stro-1/CD44+ progenitor cells in the myometrium are the likely tumor initiating cells. Stro-1 is a marker for mesenchymal stem cells (MSCs) and the big question remains whether they are stem cells or just stromal cells! The term ‘mesenchymal stem cell’ was coined in late 1980s by Arnold Caplan. He has recently modified the term to ‘medicinal signalling cell’ that are in no ways connected to stem cells. MSCs exist in vitro and arise in vivo from pericytes lining the blood vessels in various organs reviewed in [25]. We have earlier reported that MSCs invariably express cytoplasmic OCT-4. Furthermore, a sub-group of small, spherical cells expressing nuclear OCT-4A exists as a sub-group among MSCs and in both Wharton’s jelly from umbilical cord tissue and in primary cultures of bone marrow [26]. VSELs with nuclear OCT-4 evidently differentiate into mesenchymal stem cells with cytoplasmic OCT-4. Taichman et al. [27] have also reported that VSELs are at top of hierarchy for both mesenchymal and hematopoietic stem cells. Thus in the uterus also, rather than MSCs, it is the VSELs with OCT-4A that most likely expand and form fibroids. Ono et al. [28] reported higher expression of OCT-4A (but not cytoplasmic OCT-4B) in myometrial SP cells isolated from human myometrial tissue from 18 patients undergoing hysterectomy. They had concluded that OCT-4A positive cells could be involved in uterine biology as well as in pathology. Similar to our observations that P had a more prominent effect compared to estrogen on myometrium, available published literature also suggests a crucial role of progesterone pathways in the pathophysiology of uterine fibroids by use of selective progesterone receptor modulators reviewed in [29].

To conclude, the present mouse study reveals the presence of nuclear OCT-4A positive VSELs in the uterine perimetrium and implicates them as possible tumor initiator cells (TICs) rather than mesenchymal cells as suggested by others. A shift in focus towards stem cells for their role in uterine biology and pathology will provide newer therapeutic targets and also deeper understanding of the specific etiology of uterine fibroids. Further studies are required in the area and results need to be confirmed by others working in the field.

## Abbreviations

E: Estrogen
FSH: Follicle stimulating hormone
OCT-4A: Nuclear transcription factor crucial to define pluripotent state of a cell along with other markers
OCT-4B: Alternatively spliced cytoplasmic OCT-4 with no biological function
P: Progesterone
PCNA: Proliferating cell nuclear antigen
VSELs: Very small embryonic-like stem cells
